# Adrenomedullin and endothelin-1 are associated with myocardial injury and death in septic shock patients

**DOI:** 10.1186/s13054-016-1361-y

**Published:** 2016-06-09

**Authors:** Oscar H. M. Lundberg, Lill Bergenzaun, Jörgen Rydén, Mari Rosenqvist, Olle Melander, Michelle S. Chew

**Affiliations:** Department of Intensive- and perioperative care, Skåne University Hospital Malmö, Inga Marie Nilssons gata 47, S-205 02 Malmö, Sweden; Department of Infectious diseases, Skåne University Hospital Malmö, Ruth Lundskogs gata 3, S-205 02 Malmö, Sweden; Department of Internal medicine, Skåne University Hospital Malmö, 205 02 Malmö, Sweden; Lund University Institute of Clinical Sciences, Malmö, Sweden; Department of Anesthesiology and Intensive Care, Linköping University, S-58185 Linköping, Sweden; Department of Medical and Health Sciences, Linköping University, S-58185 Linköping, Sweden

**Keywords:** Sepsis, Shock, Adrenomedullin, Endothelin-1, High-sensitivity troponin, Echocardiography, Myocardial injury, Mortality, Likelihood ratio

## Abstract

**Background:**

Adrenomedullin and endothelin-1 are hormones with opposing effects on the cardiovascular system. Adrenomedullin acts as a vasodilator and seems to be important for the initiation and continuation of the hyperdynamic circulatory response in sepsis. Endothelin-1 is a vasoconstrictor and has been linked to decreased cardiac performance. Few studies have studied the relationship between adrenomedullin and endothelin-1, and morbidity and mortality in septic shock patients. High-sensitivity troponin T (hsTNT) is normally used to diagnose acute cardiac injury but is also prognostic for outcome in intensive care. We investigated the relationship between mid-regional pro-adrenomedullin (MR-proADM), C-terminal pro-endothelin-1 (CT-proET-1), and myocardial injury, measured using transthoracic echocardiography and hsTNT in septic shock patients. We were also interested in the development of different biomarkers throughout the ICU stay, and how early measurements were related to mortality. Further, we assessed if a positive biomarker panel, consisting of MR-proADM, CT-proET-1, and hsTNT changed the odds for mortality.

**Methods:**

A cohort of 53 consecutive patients with septic shock had their levels of MR-proADM, CT-proET-1, hsTNT, and left ventricular systolic functions prospectively measured over 7 days. The relationship between day 1 levels of MR-proADM/CT-proET-1 and myocardial injury was studied. We also investigated the relationship between biomarkers and early (7-day) and later (28-day) mortality. Likelihood ratios, and pretest and posttest odds for mortality were calculated.

**Results:**

Levels of MR-proADM and CT-proET-1 were significantly higher among patients with myocardial injury and were correlated with left ventricular systolic dysfunction. MR-proADM and hsTNT were significantly higher among 7-day and 28-day non-survivors. CT-proET-1 was also significantly higher among 28-day but not 7-day non-survivors. A positive biomarker panel consisting of the three biomarkers increased the odds for mortality 13-fold to 20-fold.

**Conclusions:**

MR-proADM and CT-proET-1 are associated with myocardial injury. A biomarker panel combining MR-proADM, CT-proET-1, and hsTNT increases the odds ratio for death, and may improve currently available scoring systems in critical care.

## Background

Circulatory failure is one of the most severe manifestations of early sepsis. Whilst numerous studies have investigated novel biomarkers to diagnose and risk-stratify patients with sepsis, none have become universally accepted and few have focused on the circulatory system per se. As septic shock still accounts for an unacceptable number of deaths in the critically ill, we reasoned that a biomarker strategy using a combination of clinical, biochemical, and physiological parameters focusing on the circulatory system may be one way of stratifying very high-risk patients.

Endothelial activation is a hallmark of sepsis and thought to play a key role in the pathophysiology of septic shock. In this regard, three novel biomarkers have been described that may have contributory and/or predictive roles in the development of circulatory failure – mid-regional pro-adrenomedullin (MR-proADM), C-terminal pro-endothelin-1 (CT-proET-1), and high-sensitivity troponin T (hsTNT).

Adrenomedullin (ADM) is a 52-amino acid peptide hormone, which is associated with cardiovascular, endocrine, and renal mechanisms that control fluid and electrolyte homeostasis [1]. ADM acts as a vasodilator, decreases peripheral vascular resistance, and increases cardiac output [2, 3]. ADM also decreases capillary hyperpermeability during septic shock [4, 5]. Because of the instability of the peptide, it has been shown that measurements of the mid-regional portion of the precursor peptide pro-adrenomedullin, is more suitable for clinical practice [6]. Few clinical studies have described ADM in septic shock. In the largest study to date, Guignant et al. [7] showed that increased plasma MR-proADM was associated with 28-day mortality.

Endothelin-1 (ET-1) is a 21-amino acid peptide, which acts as a potent vasoconstrictor and has mitogenic effects on smooth muscle cells. ET-1 has been shown to be involved in multiple physiological functions related to the nervous, renal, cardiovascular, respiratory, gastrointestinal, and endocrine systems [8]. Because of its short half-life (1–7 minutes) [8, 9], and almost total clearance from the blood stream by pulmonary passage, CT-proET-1 has been found to stoichiometrically measure ET-1 [9].

Cardiac troponin (cTn) is the preferred marker of myocardial ischemia and injury [10]. New high-sensitivity troponin assays have, by detecting extremely low levels, been associated with conditions other than myocardial infarction and predict worse outcome in intensive care [10–16]. As both ADM and ET-1 are potent vasoactive factors it is also plausible that they may be associated with myocardial dysfunction in sepsis [17–19]. This has been sparsely investigated in intensive care.

The aim of this study was to test whether MR-proADM and CT-proET-1 are associated with myocardial injury, measured using transthoracic echocardiography and hsTNT in patients with septic shock. We were also interested in the dynamics of MR-proADM, CT-proET-1, and hsTNT throughout the ICU stay, and how early measurements (day 1) were related to early mortality (day 7) and later mortality (day 28). Further, we assessed whether a positive biomarker panel, consisting of MR-proADM, CT-proET-1, and hsTNT changes the odds of mortality.

## Methods

The study was approved by the Regional Ethical Review Board, Lund, Sweden (Dnr.187/2005). Informed consent was sought either from the patient or, if not possible, from the patient’s next of kin. The study design comprised a single-center, prospective observational cohort of critically ill patients admitted to the mixed-bed ICU of Skåne University Hospital, Malmö, Sweden. Data collection lasted up to a maximum of 7 days, or until ICU discharge, or death if either occurred before 7 days. Early (7-day) and later (28-day) mortality was measured. Fifty-five consecutive patients with septic shock were included between year 2005 and 2007. Septic shock was defined according to the criteria published by Dellinger et al. [20]. Exclusion criteria were pregnancy, inherited abnormalities of coagulation, fibrinolytic therapy, compromised immunity or a “Do not attempt resuscitation” order. Patients could be included only once. All patients were initially treated according to international guidelines for the management of sepsis and septic shock [21]. After the initial resuscitation period, fluids were given at the treating clinician’s discretion. Acute physiology and chronic health evaluation (APACHE) II scores were calculated at admission and sequential organ failure assessment (SOFA) scores were calculated daily.

### Biochemical analyses

Blood samples were collected from an indwelling arterial line. MR-proADM and CT-proET-1 were measured four times on day 1 (first sample within 6 hours of arrival to the ICU), twice on day 2, and thereafter once daily until ICU discharge, death or end of study. HsTNT was measured twice on day 1 (first sample within 12 hours of arrival to the ICU) and thereafter once daily until ICU discharge, death or end of study. The daily values of all biomarkers were averaged to give a single representative value for that day. The blood samples were sent to the local clinical chemistry laboratory, Skåne University Hospital, Malmö, Sweden, where they were centrifuged, frozen at −80 °C, and stored.

MR-proADM and CT-proET-1 were batch-analyzed using a sandwich immunoassay (BRAHMS GmbH/ThermoFischer Scientific, Henningsdorf, Germany). In the general population, 90 % of measurements of MR-proADM are below 0.55 nmol/L [22] and the 99^th^ percentile of CT-proET-1 in a healthy population is 72.9 pmol/L [9]. The analytical detection limits of MR-proADM and CT-proET-1 were 0.08 nmol/L and 4.3 pmol/L. HsTNT was measured using an immunoassay (Cobas e601, Roche Diagnostics GmbH, Penzberg, Germany) [23]. The measurement range is 3–10,000 ng/L and the upper reference limit (99^th^ percentile) is 14 ng/L in healthy volunteers.

### Echocardiography

TTE examinations were performed within 12 hours of inclusion for the evaluation of left ventricular (LV) systolic function. Images were acquired using a Hewlett- Packard Sonos 5500 (Andover, MA, USA) scanner and a 3 MHz transducer. Two-dimensional (2D) imaging examinations were performed in the standard apical four-chamber and two-chamber views. Tissue harmonic imaging was used to enhance 2D image quality. Parameters of LV systolic function (left ventricular ejection fraction (LVEF), mitral annular plane systolic excursion (MAPSE), peak systolic tissue Doppler velocity imaging (TDIs) and velocity time integral in the left ventricular outflow tract (LVOT VTI)) were acquired as described previously [24].

### Myocardial injury

Myocardial injury was defined as an hsTNT value ≥15 ng on day 1 and at least two of the following echocardiographic parameters on day 1: LVEF ≤50 %, MAPSE ≤12 mm, or TDIs ≤7.5 cm/sec.

### Statistics

A sample size of 46 patients was required to detect a posttest myocardial injury risk of 0.75, assuming a baseline risk of 0.3. This was calculated as a test of proportions with a two-tailed α value of 0.05 and β of 0.8, with a continuity correction applied. As we expected dropouts we arbitrarily chose to increase the sample size to a convenience sample of 55 patients.

Data are presented as median (interquartile range), percentages or absolute values. IBM SPSS Statistics version 22 was used for statistical calculations. For non-normally distributed variables we used non-parametric tests. Missing values were considered as randomly missing and were not adjusted for. Spearman’s rank correlation was calculated to test correlation between two variables, and for differences between two groups we used the Mann-Whitney *U* test. Categorical data were analyzed with Fisher’s exact test. We used Holm’s procedure to adjust for multiple testing. Receiver operating characteristic (ROC) curve analysis was performed with calculation of maximal area under the curve (AUC). Youden’s index was used to define optimal cutoff values. The positive predictive value (PPV) and negative predictive value (NVP) were calculated. For the evaluation of the diagnostic accuracy of each biomarker, we calculated the positive likelihood ratio (LR+) and negative likelihood ratio (LR-), where LR+ is the sensitivity/(1 – specificity) and LR- is (1 – sensitivity)/specificity. Confidence intervals (CI) were calculated for each likelihood ratio. The pretest odds of mortality is given by *P*/(1 – *P*), where *P* is the probability of the mortality in the current study cohort. The posttest odds, given a positive test, are the product of the LR+ and pretest odds, whereas the posttest odds, given a negative test, are the product of the LR- and the pretest odds.

## Results

Two patients were excluded due to lack of written consent leaving 53 patients included in the study. Three patients had missing hsTNT and six patients had missing echocardiographic data. The patients’ medical histories divided them into surgical (n = 16) and medical (n = 37) cases. The 7-day and 28-day mortality was 19 % and 28 %, respectively. Survivors tended to be younger, and had lower APACHE II and SOFA scores at admission as shown in Table 1.

**Table 1:** Baseline characteristics of all patients and according to survival

		Mortality at 28 days			Mortality at 7 days		
	All	Survivors	Non-survivors	*P* value	Survivors	Non-survivors	*P* value
	(n = 53)	(n = 38)	(n = 15)		(n = 43)	(n = 10)	
Age, years	65 (20)	60 (22)	72 (8)	0.007	61 (19)	76 (8)	0.026
APACHE II, score	24 (10)	23 (11)	28 (14)	0.026	24 (11)	29 (10)	0.015
SOFA score, admission	12 (5)	11 (4)	14 (2)	0.002	11 (4)	14 (3)	0.002
Body mass index, kg/m^2^	26 (5)	27 (7)	24 (4)	0.008	26 (7)	24 (4)	0.094
Gender (male/female), *n*	37/16	26/12	11/4	1	30/13	7/3	1
Medical/surgical, *n*	37/16	26/12	11/4	1	30/13	7/3	1

*APACHE* acute physiology and chronic health evaluation, *SOFA* sequential organ failure assessment

### Temporal development of biomarkers

**Fig. 1:**
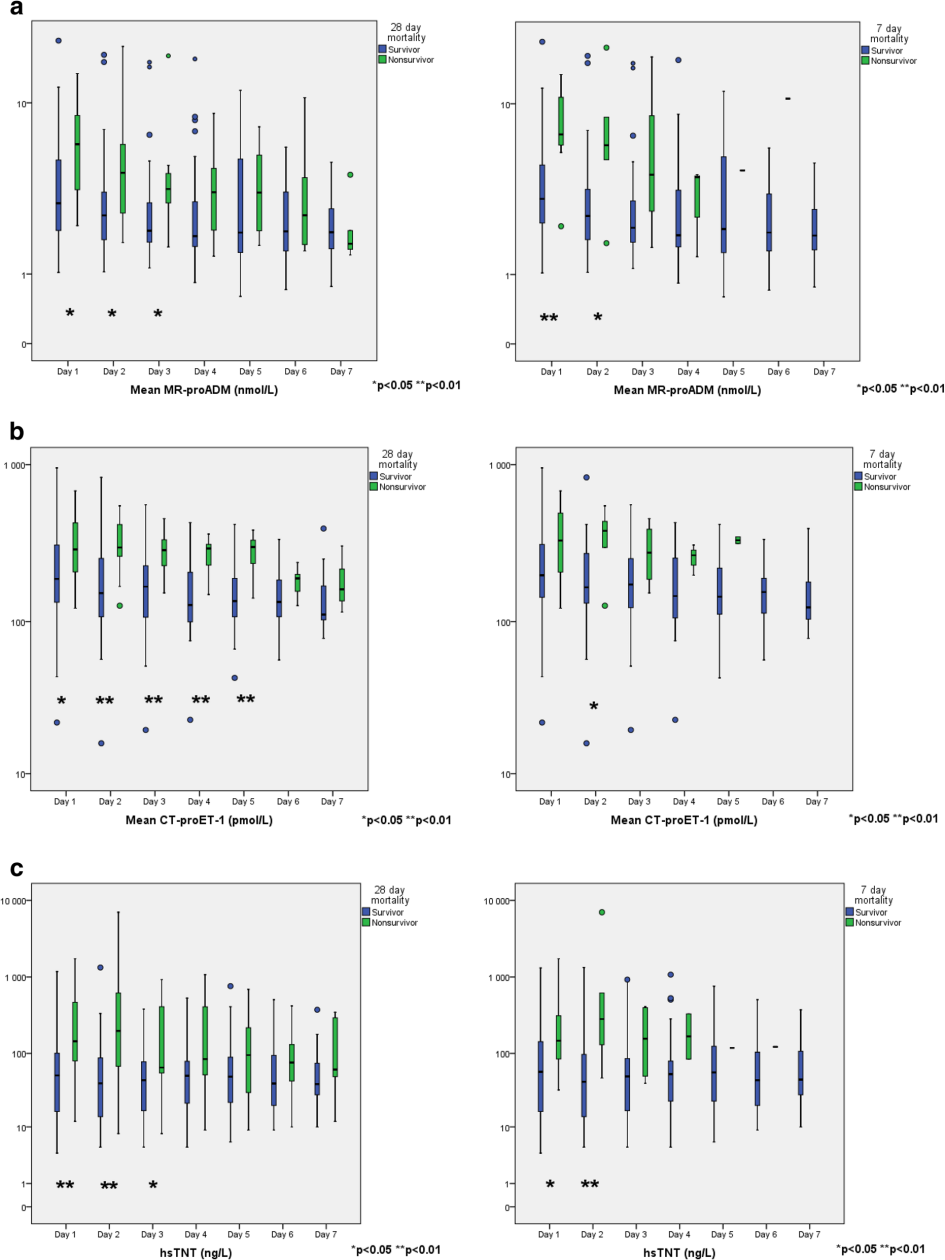
Temporal development of MR-proADM, CT-proET-1 and hsTNT

Figure 1 (a-c) shows the temporal development of MR-proADM, CT-proET-1, and hsTNT according to short (7-day) and longer-term (28-day) mortality. Non-survivors generally had higher values of all biomarkers over the 7-day period.

### Relationship between MR-proADM, CT-proET-1, and myocardial injury

There was statistically significant inverse correlation between MR-proADM measured on day 1 and two of the four echocardiographic markers of LV systolic dysfunction, MAPSE and LVOT VTI. Day 1 CT-proET-1 concentrations were inversely correlated to all LV systolic function parameters (*ρ* = –0.43 to –0.48, *p* = 0.001–0.003). Both MR-proADM and CT-proET-1 were also correlated with hsTNT (*ρ* = 0.38, *p* = 0.007 and *ρ* = 0.40, *p* = 0.004, respectively). Both biomarkers were significantly correlated with each other (*ρ* = 0.68, *p* ≤ 0.001), age, and creatinine (see Table 2).

**Table 2:** Correlation between MR-proADM/CT-proET-1 and echocardiographic markers of left ventricular systolic function, hsTNT, age, and creatinine

		LVEF	MAPSE	TDIs	LVOT VTI	hsTNT	Age	Creatinine
MR-proADM	Correlation coefficient ρ	-0.139	-0.320	-0.142	-0.310	0.376	0.342	0.741
	p value	0.351	0.029	0.342	0.036	0.007*	0.012	>0.001*
CT-proET-1	Correlation coefficient ρ	-0.439	-0.479	-0.430	-0.437	0.396	0.385	0.524
	p value	0.002*	0.001*	0.003*	0.002*	0.004*	0.004*	>0.001*

*MR-proADM* mid-regional pro-adrenomedullin, *CT-proET-1* C-terminal pro-endothelin-1, *LVEF* left ventricular ejection fraction, *MAPSE* mitral annular plane systolic excursion, *TDIs* peak systolic tissue Doppler velocity imaging, LVOT VTI velocity time integral in the left ventricle outflow tract, *hsTNT* high-sensitivity troponin T. *P value lower than adjusted alpha after Holm’s procedure for multiple testing

Twenty-six patients had myocardial injury defined as above, and these patients had significantly higher levels of MR-proADM and CT-proET-1 (*p* = 0.007 and *p* < 0.001, respectively) (see Table 3).

**Table 3:** Biomarkers related to myocardial injury and mortality

	Myocardial injury			Mortality at 28 days			Mortality at 7 days		
	No (n = 21)	Yes (n = 26)	*P* value	Survivors	Non-survivors	*P* value	Survivors	Non-survivors	*P* value
MR-proADM	2.5 (2.4)	5.2 (5.8)	0.007	3.0 (3.4)	6.3 (6.7)	0.010	3.3 (2.9)	7.1 (5.2)	0.002*
CT-proET-1	153 (111)	324 (238)	<0.001*	188 (183)	289 (247)	0.027	198 (172)	332 (319)	0.088
hsTNT	-	-	-	51 (85)	143 (444)	0.007	57 (126)	146 (388)	0.033
Creatinine	-	-	-	138 (150)	182 (131)	0.211	122 (129)	200 (132)	0.048

*MR-proADM* mid-regional pro-adrenomedullin, *CT-proET-1* C-terminal pro-endothelin-1, *hsTNT* high-sensitivity troponin T. *P value lower than adjusted alpha after Holm’s procedure for multiple testing

### Relationship between biomarker concentrations on day 1 and mortality

The day-1 mean plasma levels according to 7-day and 28-day mortality are displayed in Table 3. MR-proADM, CT-proET-1 and hsTNT were significantly higher among patients who did not survive 28 days. MR-proADM and hsTNT but not CT-proET-1 were higher in patients who did not survive 7 days.

### Odds and predictive values for single and combined biomarkers

Table 4 shows the AUC and cutoff values from the ROC curves, and the corresponding PPVs and NVPs. The cutoff values were used when calculating the LR and odds shown in Table 5. The LR+ for MR-proADM was 4.3 when calculated for 7-day mortality. When MR-proADM and CT-proET-1 were combined the LR+ increased. The highest values for the LR+ were obtained when combining all three biomarkers – the difference between the pretest and posttest odds was up to 20-fold (0.35–6.97) for 28-day mortality and 13-fold (0.19–2.49) for 7-day mortality. When MR-proADM and CT-proET-1 were combined the difference between the pretest and posttest odds was 12-fold (1.24–14.9) for myocardial injury.

**Table 4:** Area under the curve (AUC), cutoff and positive predictive value/negative predictive value (PPV/NPV)

	Myocardial injury					Mortality at 28 days					Mortality at 7 days				
	Cutoff	AUC	Sensitivity	Specificity	PPV/NPV	Cutoff	AUC	Sensitivity	Specificity	PPV/NPV	Cutoff	AUC	Sensitivity	Specificity	PPV/NPV
MR-proADM	4.6 nmol/L	0.729	0.577	0.810	0.79/0.61	3.5 nmol/L	0.730	0.8	0.605	0.44/0.88	5.5 nmol/L	0.823	0.9	0.791	0.5/0.97
CT-proET-1	209 pmol/L	0.855	0.808	0.810	0.81/0.76	206 pmol/L	0.696	0.8	0.579	0.41/0.88	269 pmol/L	0.674	0.7	0.651	0.32/0.90
hsTNT	-	-	-	-	-	114 ng/L	0.752	0.692	0.784	0.53/0.88	114 ng/L	0.74	0.75	0.738	0.35/0.94
APACHE II	-	-	-	-	-	27	0.696	0.667	0.737	0.5/0.88	27	0.744	0.8	0.721	0.4/0.94

*MR-proADM* mid-regional pro-adrenomedullin, *CT-proET-1* C-terminal pro-endothelin-1, *hsTNT* high-sensitivity troponin T, *APACHE* acute physiology and chronic health evaluation

**Table 5:** Likelihood ratios (LR) (95 % CI) and odds

	Myocardial injury				Mortality at 28 days				Mortality at 7 days			
	LR+	Given positive test pre/posttest odds	LR-	Given negative test pre/posttest odds	LR+	Given positive test pre/posttest odds	LR-	Given negative test pre/posttest odds	LR+	Given positive test pre/posttest odds	LR-	Given negative test pre/posttest odds
MR-proADM	3.03	1.24/3.76	0.52	1.24/0.64	2.03	0.39/0.79	0.33	0.39/0.13	4.3	0.23/0.99	0.13	0.23/0.03
	(1.18, 7.76)		(0.32, 0.86)		(1.269, 3.236)		(0.116, 0.939)		(2.32, 7.97)		(0.02, 0.82)	
CT-proET-1	3.39	1.24/4.20	0.25	1.24/0.31	1.79	0.39/0.70	0.46	0.39/0.14	2.01	0.23/0.46	0.46	0.23/0.11
	(1.54, -7.46)		(0.11, 0.57)		(1.158, 2.762)		(0.233, 0.987)		(1.13, 3.57)		(0.17, 1.22)	
hsTNT	-	-	-	-	3.20	0.35/1.12	0.39	0.35/0.14	2.86	0.19/0.54	0.34	0.19/0.07
					(1.570, 6.529)		(0.171, 0.903)		(1.50, 5.47)		(0.10, 1.14)	
MR-proADM and CT-proET-1	12	1.24/14.9	0.44	1.24/0.55	2.14	0.39/0.84	0.27	0.39/0.10	5.02	0.23/1.15	0.17	0.23/0.04
	(1.74, 84)		(0.28, 0.70)		(1.25, 3.67)		(0.07, 1.01)		(1.95, 9.47)		(0.02, 1.04)	
MR-proADM, CT-proET-1 and hsTNT	-	-	-	-	19.92	0.35/6.97	NA*	NA*	13.13	0.19/2.49	NA*	NA*
					(2.70, 146.88)				(3.06, 56.24)			

*MR-proADM* mid-regional pro-adrenomedullin, *CT-proET-1* C-terminal pro-endothelin-1, *hsTNT* high-sensitivity troponin T. NA, not analyzed. *It was not possible to calculate the LR- for all three biomarkers combined, because none of the patients with low values died

## Discussion

### Biomarkers and myocardial injury

In this exploratory study we demonstrated significant relationships between MR-proADM/CT-proET-1 and myocardial injury. The relationship was strongest and most consistent with CT-proET-1. This finding supports a biologically plausible relationship as both pro-hormones are strongly vasoactive and may play key roles in sepsis-associated myocardial injury. Indeed, we demonstrated significant associations between both pro-hormones and hsTNT and echocardiographic markers of LV systolic dysfunction.

In epidemiological studies, increased MR-proADM has been associated with poor cardiovascular outcomes [22, 25, 26]. In sepsis there is upregulation of ADM expression [27, 28] and ADM seems to be important for the initiation and continuation of hyperdynamic shock in animal models [4, 5, 29–31]. Importantly, the administration of anti-ADM antibodies prevents the hyperdynamic response [27] and seems beneficial to survival [32, 33], while exogenous ADM prevents and reverses hypodynamic circulation and pulmonary hypertension, and reduces endothelial hyperpermeability in experimental models of septic shock [4, 5, 30, 34], suggesting possibilities for therapeutic intervention. In this study we found only moderate correlation between MR-proADM and two of four echocardiographic markers of reduced LV systolic function. Despite this there was strongly significant correlation between proADM and hsTNT concentrations, which could suggest a role of this pro-hormone in cardiac injury.

Experimental and clinical studies link increased ET-1 levels to decreased cardiac performance [17, 19, 35–38]. This is supported by our findings of highly significant correlation between CT-proET-1 levels and all echocardiographic markers of reduced LV systolic function, and hsTNT. The results of these studies appear paradoxical to earlier experimental data showing positive inotropic effects of ET-1 [39, 40]. Thus, the role of ET-1 is still unclear and seems related to the balance between receptor types.

Antagonism of endothelin pathways has been explored in a number of experimental settings, and its effects during septic shock are areas worth exploring [35–37, 41–43]. To our knowledge, there is only one other study investigating the relationship between cardiac function and CT-proET-1 in patients with septic shock. Furian et al. [17] demonstrated significant association between CT-proET-1 and echocardiographic markers of left and right ventricular dysfunction, but did not describe biochemical markers of myocardial injury. Our findings highlight the importance of CT-proET-1 in cardiac dysfunction measured using echocardiography and cardiac troponins, and in mortality. Importantly, the LR- of 0.25 indicates that CT-proET-1 is useful for ruling out myocardial injury. Taken together, our results indicate that the combination of MR-proADM and CT-proET-1 might be a useful supplement for the diagnosis of myocardial injury, as shown by a LR+ of 12.

### Biomarkers and mortality

We have shown that increased concentrations of MR-proADM, CT-proET-1, and hsTNT are increased in non-survivors of septic shock, supporting the results of earlier studies [7, 11, 16, 19, 44–46]. MR-proADM and hsTNT seem to be more important determinants of both short-term and longer-term outcome, whereas CT-proET-1 seems to be most significant for longer-term mortality with higher concentrations detected in non-survivors on days 2–5 (Fig. 1b). When considered as a pair, CT-proET-1 and MR-proADM increased the odds for mortality twofold to fivefold. When a combined panel of all three biomarkers were positive, the posttest odds for mortality increased 13-fold to 20-fold.

ProADM and proET-1 are especially attractive biomarkers in septic shock because they are both endothelium-derived pro-hormones and their end products have important vasoregulatory opposing effects. As suggested by Scheutz and colleagues [45] it is plausible that the net balance between the hormones is of significance for clinical outcome. Increased concentrations of ADM and ET-1 have been described in patients with systemic inflammatory response syndrome (SIRS) [47] and septic shock [6, 7, 17, 29, 44–46, 48], and appear to be related to severity and mortality, but dynamic evaluations and their significance for short-term and long-term mortality in patients with shock are poorly investigated. Herein we demonstrated that concentrations of both pro-hormones are higher in non-survivors, particularly during the first 3 days of ICU admission (see Fig. 1).

In line with our results, Guignant et al. reported higher initial levels of proADM among non-survivors of septic shock. Further, the combination of proADM with a vasoconstrictor biomarker, pro-vasopressin, was better for prediction of 28-day mortality when assessed at day 1–2 than the SOFA score and simplified acute physiology score (SAPS) II [7]. Similarly, in a cohort of critically ill patients with sepsis, Christ-Crain et al. found a significantly higher level of proADM among intensive care unit (ICU) non-survivors [46]. They reported an optimal cutoff value of 3.9 nmol/L for MR-proADM, resembling the optimal cutoff of 3.5 nmol/L identified in this study for 28-day mortality. The optimal cutoff identified by Guignant et al. was also in this range (5 nmol/L) [7]. Taken together, these findings support proADM as a useful predictor of mortality.

Our results for ET-1 are different to those reported previously, where no differences between survivors and non-survivors were shown [45, 49]. There may be several explanations for this. First, our patients were severely ill with higher illness severity scores than in previous studies. The median day 1 SOFA and APACHE II scores were 12 and 24, respectively, and all 53 patients were in shock despite fluid resuscitation. Second, we used 7-day and 28-day mortality as outcome parameters, in contrast to in-hospital mortality as used in some of the other studies. Third, we collected blood 6-hourly in the first 24 hours, and used average daily values in an attempt to capture average values for each patient every day. In comparison, Scheutz et al. collected a single sample within 24 hours of ICU admission. Guignant et al. collected a single sample within 48 hours of ICU admission and had a substantial number of missing values. These reflect difficulties in the conduct of clinical studies but may be of significance, as measuring biomarker levels at an early stage, i.e., when the patient is most unstable, may reveal important information about the state of the cardiovascular system. It also allows the possibility of early intervention and disease staging.

Although elevated cTn is most commonly used for the diagnosis of MI [50], increased cTns are commonly seen in patients with septic shock without MI and are independent predictors of mortality [11–15]. Recent studies suggest that high-sensitivity assays may add to risk assessment and prediction models [11, 16]. Our study confirms the importance of hsTNT for the outcome of patients with septic shock. When used as an indicator of injury along with echocardiographic parameters, it may potentially be used to stratify risk and monitor treatment. Both alone, but especially when used in a biomarker panel with MR-proADM and CT-proET-1, hsTNT increased the posttest odds ratio of mortality by 13-fold to 20-fold.

It remains to be seen whether this biomarker panel ultimately improves current risk prediction models in critical care. Another potential area of investigation is the use of these biomarkers as a basis for selection of patients for interventional studies, or as pharmacodynamic markers for cardiac dysfunction.

### Limitations

This paper has several limitations. This study was designed to be exploratory in nature and the findings here confirm associations between biomarkers and outcome, and refrains from any conclusions on causality. The limited number of outcome events does not allow adequate power for multivariate analysis. As a rule-of-thumb 10 outcome events would be required for each multivariate variable [51], thus, future studies investigating the prognostic potential of these biomarkers should be planned with this in mind. While we realize the limitations of this type of monocenter investigation, in particular the risk of overestimation of effect size, we believe that our study contributes new information to a hitherto under-investigated area. Second, although we defined ICU admission as a starting point for this study, patients have had variable times to presentation, different degrees of shock and variable responses to fluid resuscitation, making the material potentially heterogeneous. As dynamic changes in biomarker levels may be important, particularly early in the course of septic shock, we attempted to capture these changes by measuring up to four times during the first 24 hours, and twice daily during ensuing days. Closer sampling times may have revealed different results. We have no data on right ventricular echocardiographic parameters. As almost all components of the endothelin system are upregulated in pulmonary hypertension [8], and right ventricular dysfunction is common in septic shock, it is plausible that high levels of CT-proET-1 could correlate with right ventricular dysfunction. Because of the lack of a universal definition of myocardial injury, our definition was arbitrary but chosen on the basis of previous studies [23, 52–56]. As premorbid echocardiographic data were not available, we cannot exclude that some patients suffered from co-existing myocardial dysfunction that was unrelated to sepsis.

## Conclusion

Our study shows that MR-proADM and CT-proET-1 are associated with myocardial injury and dysfunction. It also supports the concept of a composite biomarker panel for adverse outcome prediction or risk stratification as proposed in earlier studies in patients with sepsis. We found that this particular combination of MR-proADM, CT-proET-1 and hsTNT markedly increased the posttest odds of death in a population of severely ill patients.

## Key messages

- MR-proADM and CT-proET-1 are correlated with myocardial injury in patients with septic shock
- A positive biomarker panel consisting of MR-proADM, CT-proET-1, and hsTNT increases the odds of both short-term and longer-term mortality

## Abbreviations

2D, two-dimensional; ADM, adrenomedullin; APACHE II, acute physiology and chronic health evaluation II; AUC, area under the curve; BMI, body mass index; CI, confidence interval; cTn, cardiac troponin; CT-proET-1, C-terminal pro-endothelin-1; ET-1, endothelin-1; hsTNT, high-sensitivity troponin T; ICU, intensive care unit; LR-, negative likelihood ratio; LR+, positive likelihood ratio; LV, left ventricle; LVOT VTI, velocity time integral in the left ventricle outflow tract; MAPSE, mitral annular plane systolic excursion; MI, myocardial infarction; MR-proADM, mid-regional pro-adrenomedullin; NPV, negative predictive value; PPV, positive predictive value; ROC, receiver operating characteristics curve; SAPS II, simplified acute physiology score II; SIRS, systemic inflammatory response syndrome; SOFA, sequential organ failure assessment ; TDIs, peak systolic tissue Doppler velocity imaging
